# The complete chloroplast genome sequence of *Ehrharta erecta* Lam. (Poaceae)

**DOI:** 10.1080/23802359.2021.1931503

**Published:** 2021-05-24

**Authors:** Wei-Cai Song, Qi Feng, Yun-Jiao Zhang, Chao Shi

**Affiliations:** a College of Marine Science and Biological Engineering, Qingdao University of Science and Technology, Qingdao, China; b Kunming Medical University Haiyuan College, Kunming, China; c Plant Germplasm and Genomics Center, Germplasm Bank of Wild Species in Southwest China, Kunming Institute of Botany, The Chinese Academy of Sciences, Kunming, China

**Keywords:** Poaceae, *Ehrharta erecta*, chloroplast genome, phylogenetic relationship

## Abstract

The complete chloroplast (cp) genome of *Ehrharta erecta* was sequenced and assembled for the first time. In this study, The total genome size is 134,511 bp in length and demonstrates a typical quadripartite structure containing a large single copy (LSC, 95,227 bp) and a small single copy (SSC, 12,306 bp), separated by a pair of inverted repeats (IRa, IRb) of 13,489 bp. The G + C content of this chloroplast genome was 38.76%. Gene annotation analysis identified 130 genes including 84 protein-coding genes, 38 transfer RNA, and 8 ribosomal RNA genes. The maximum-likelihood phylogenetic analysis result showed that *E. erecta* was closely related to *O. sativa* in the phylogenetic relationship.

*Ehrharta erecta* is a perennial grass of the grass family Poaceae native to South Africa and is a common invasive species in several places around the world, including Hawaii, Australia, New Zealand, the Mediterranean, and China (Frey 2005; Calvo and Moreira-Muñoz 2018). They are annual or perennial plants and can tolerate a wide range of abiotic conditions, such as drought conditions and high shade (Manea et al. 2016). *E. erecta* can reach heights of 60 cm, has 5- to 15-cm long leaves, and 6- to 20-cm-long panicle-like inflorescences with sessile to subsessile spikelets that look like beads on a necklace (Holloran et al. 2004; Grove 2012). In China, *E. erecta* are grown in large numbers and used as high-quality pasture for animals. It is important to study the chloroplast of *E. erecta* to increase yield.

Fresh leaves of *E. erecta* were collected from Panlong District, Kunming City, Yunnan Province, China (24°23′N, 102°10′E), and the voucher specimen and DNA were deposited at Qingdao University of Science and Technology (specimen code HY0812). Total genomic DNA was extracted from fresh leaves using modified CTAB (Allen et al. 2006), the high-quality DNA was sent to construct a genomic library and sequenced using the Illumina HiSeq platform in Novogene (Nanjing, China). About 1.2 Gb high quality, 2 × 150 bp pair-end reads were obtained and were used to assemble the complete chloroplast genome of *E. erecta* (Wang et al. 2018). The chloroplast genome of *E. pilosa* (Genbank accession no. MN268502) was used as a reference to assemble the complete chloroplast genome of *E. erecta* (Genbank accession no. MW013317) by NOVOPlasty4.2 (Dierckxsens et al. 2017). We also deposited the raw sequencing reads in SRA with Accession No. SRR13805805. Gene annotation was performed with the GeSeq (Michael et al. 2017) and manually corrected for codons and gene boundaries using the Sequin.

The complete chloroplast genome reported here is 134,511 bp in length and exhibits a typical quadripartite structure in, consisting of a pair of inverted repeat regions (IRa and IRb) with same length (13,489 bp) separated by the large single copy (LSC, 95,227 bp) and small single copy (SSC, 12,306 bp) regions. The overall GC content is 38.76%, and the corresponding values of the LSC, SSC and IR regions are 35.91, 32.31, and 43.41%, respectively. The chloroplast genome of *E. erecta* comprised 130 genes, including 84 protein-coding genes, 38 transfer RNA, and 8 ribosome RNA. Noticeably, nine protein-coding genes (*ndhA, rpl2, rpl16, petD, petB, rpoC1, atpF, rps16,* and *ndhB*) were disrupted by one intron, and three genes (*clpP, rps12, and ycf3*) by two.

An alignment comprising the complete chloroplast genome sequences of *E. erecta* and other 14 related taxa of Poaceae was performed in MAFFT version 7.407 (Nakamura et al. 2018; Yupeng et al. 2020). Mosel selected process in Mega version X (Kumar et al. 2018) and GTR + G + I was selected by the Akaike Information Criterion. Phylogenetic tree was constructed using maximum-likelihood (ML) method and bootstrap with 1000 times iteration using the Mega version X (Figure 1). The phylogenetic analysis results clearly showed that *E. erecta* was belonged to Poaceae and closer to *O. sativa*, these findings further enriched the phylogenetic relationship of the family Poaceae and will provide useful genetic information for promoting the evolutionary studies of Poaceae species.

**Figure 1.**
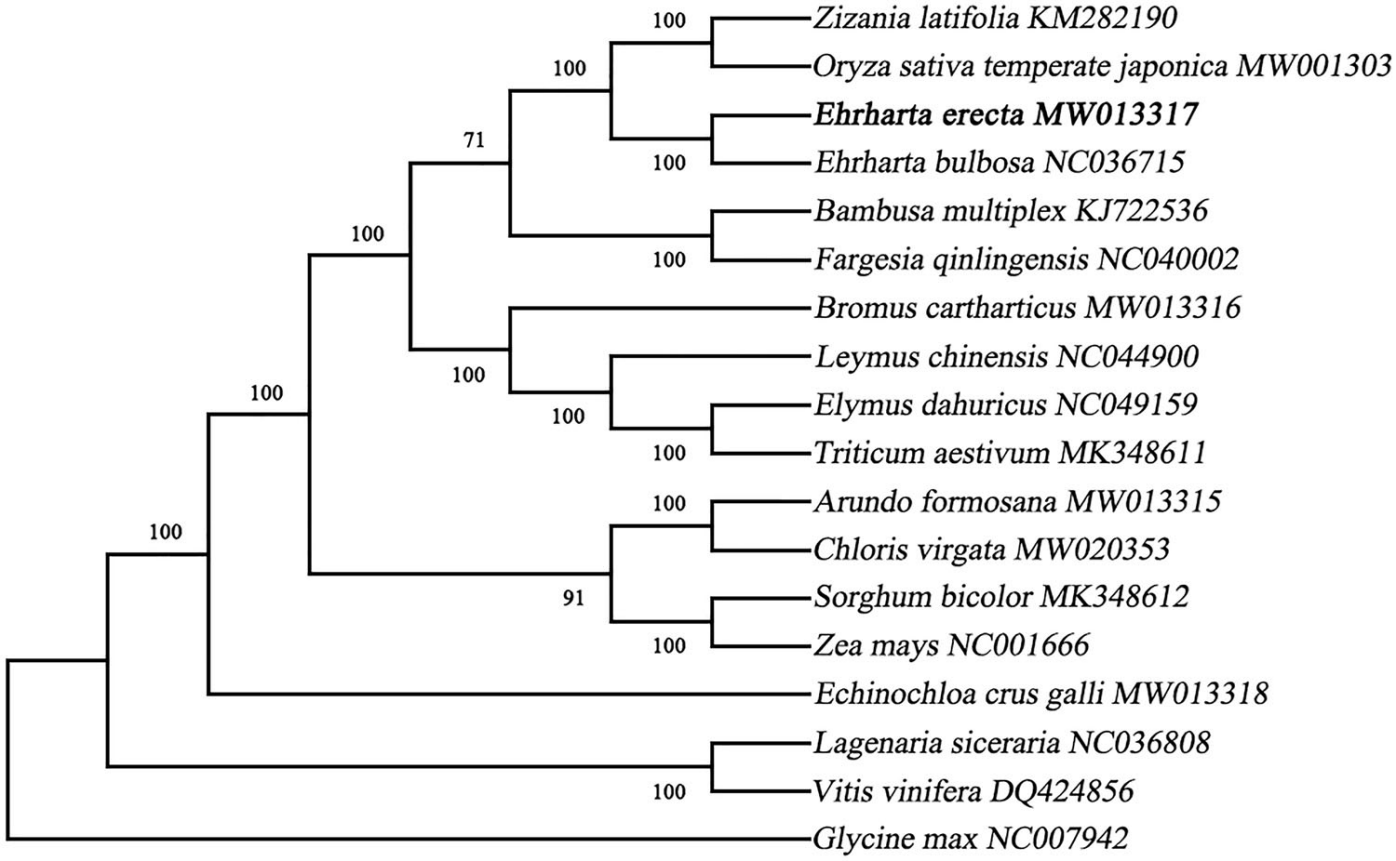
A maximum-likelihood tree illustrates the phylogenetic position of *E. erecta* among part of Poaceae species. The number on each node indicates bootstrap support value. After species is the chloroplast genome sequence login number used by GenBank.

## Data Availability

The genome sequence data that support the findings of this study are openly available in GenBank of NCBI at (https://www.ncbi.nlm.nih.gov/) under the accession no. MW013317. The associated BioProject, SRA, and Bio-Sample numbers are PRJNA705412, SRR13805805, and SAMN18087868, respectively.
